# Cerebral Cavernous Malformation: From Genetics to Pharmacotherapy

**DOI:** 10.1002/brb3.70223

**Published:** 2024-12-31

**Authors:** Zhuangzhuang Zhang, Jianwen Deng, Weiping Sun, Zhaoxia Wang

**Affiliations:** ^1^ Department of Neurology Peking University First Hospital Beijing China

**Keywords:** cerebral cavernous malformation, germline mutation, pharmacotherapy, somatic mutation

## Abstract

**Introduction:**

Cerebral cavernous malformation (CCM) is a type of cerebrovascular abnormality in the central nervous system linked to both germline and somatic genetic mutations. Recent preclinical and clinical studies have shown that various drugs can effectively reduce the burden of CCM lesions. Despite significant progress, the mechanisms driving CCM remain incompletely understood, and to date, no drugs have been developed that can cure or prevent CCM. This review aims to explore the genetic mutations, molecular mechanisms, and pharmacological interventions related to CCM.

**Methods:**

Literatures on the genetic mechanisms and pharmacological treatments of CCM can be searched in PubMed and Web of Science.

**Results:**

Germline and somatic mutations mediate the onset and development of CCM through several molecular pathways. Medications such as statins, fasudil, rapamycin, and propranolol can alleviate CCM symptoms or hinder its progression by specifically modulating the corresponding targets.

**Conclusions:**

Understanding the molecular mechanisms underlying CCM offers potential for targeted therapies. Further research into novel mutations and treatment strategies is essential for improving patient outcomes.

## Graphical abstract

*CCM1*, *CCM2*, *CCM3*, *MAP3K3*, and *PIK3CA* genes mediate CCM pathogenesis by regulating the RhoA–ROCK, MAP3K3–KLF2/4, and PIK3CA–AKT–mTOR pathways, affecting processes such as cell migration, intercellular junctions, cell proliferation, inflammation, autophagy, EndMT, angiogenesis, and oxidative stress. Therapeutic agents such as statins, propranolol, vitamin D, fasudil, and rapamycin have shown favorable efficacy.

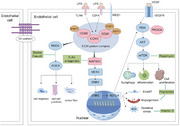

**Abbreviations**
AKT: protein kinase B
CCM: cerebral cavernous malformation
CD14: cluster of differentiation 14
EndMT: endothelial‐to‐mesenchymal transition.
ERK5: extracellular signal‐regulated kinase 5
HEG1: heart of glass homolog 1
ICAP1: integrin cytoplasmic domain‐associated protein‐1
KLF2/4: Krüppel‐like factor 2/4
LPS: lipopolysaccharide
MAP3K3: mitogen‐activated protein kinase kinase kinase 3
MEK5: mitogen‐activated protein kinase kinase 5
mTOR: mechanistic target of rapamycin
PI3K: phosphoinositide 3‐kinase
PIK3CA: phosphatidylinositol‐4,5‐bisphosphate 3‐kinase catalytic subunit alpha
RAP1: ras‐proximate‐1
RhoA: ras homolog family member A
ROCK: rho‐associated coiled‐coil containing protein kinase
smurf1: smad ubiquitination regulatory factor 1
TLR4: toll‐like receptor 4
VEGF: vascular endothelial growth factor
VEGFR: vascular endothelial growth factor receptor

## 1 Introduction

Cerebral cavernous malformation (CCM) is a structural anomaly in the central nervous system marked by multiple mulberry‐shaped dilated cavernous vessels with thin‐walled capillaries, lacking the normal brain parenchymal structure (Petersen et al. 2010; Flemming et al. 2017; Smith 2024). CCM constitutes 5%–15% of all central nervous system vascular malformations, second only to cerebral arteriovenous malformations (Dalyai et al. 2011; Haasdijk et al. 2012). Symptoms arise from hemorrhage and the expansion of abnormal blood vessels in and around the lesion, commonly presenting as focal neurological deficits, localized seizures, and severe headaches (Smith 2024; Flemming 2017; Al‐Shahi Salman et al. 2012; Flemming and Lanzino 2020).

**FIGURE 1:**
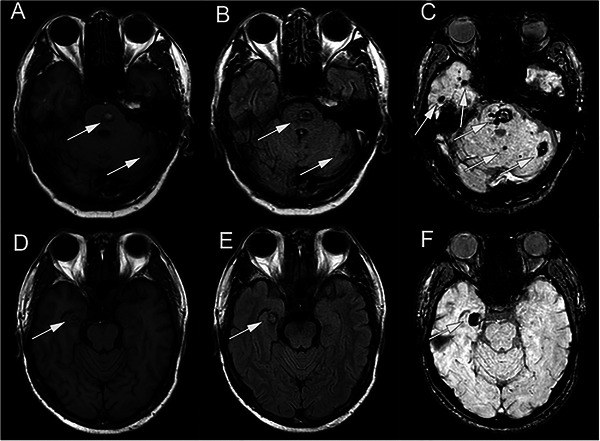
Non‐enhanced brain MRI of familial CCM and sporadic CCM. (A) T1, (B) FLAIR, and (C) SWI sequences of a familial CCM patient with multiple lesions in the pons, cerebellum, and temporal lobe (arrows). (D) T1, (E) FLAIR, and (F) SWI sequences of a sporadic CCM patient with a single lesion in the temporal lobe (arrows).

**FIGURE 2:**
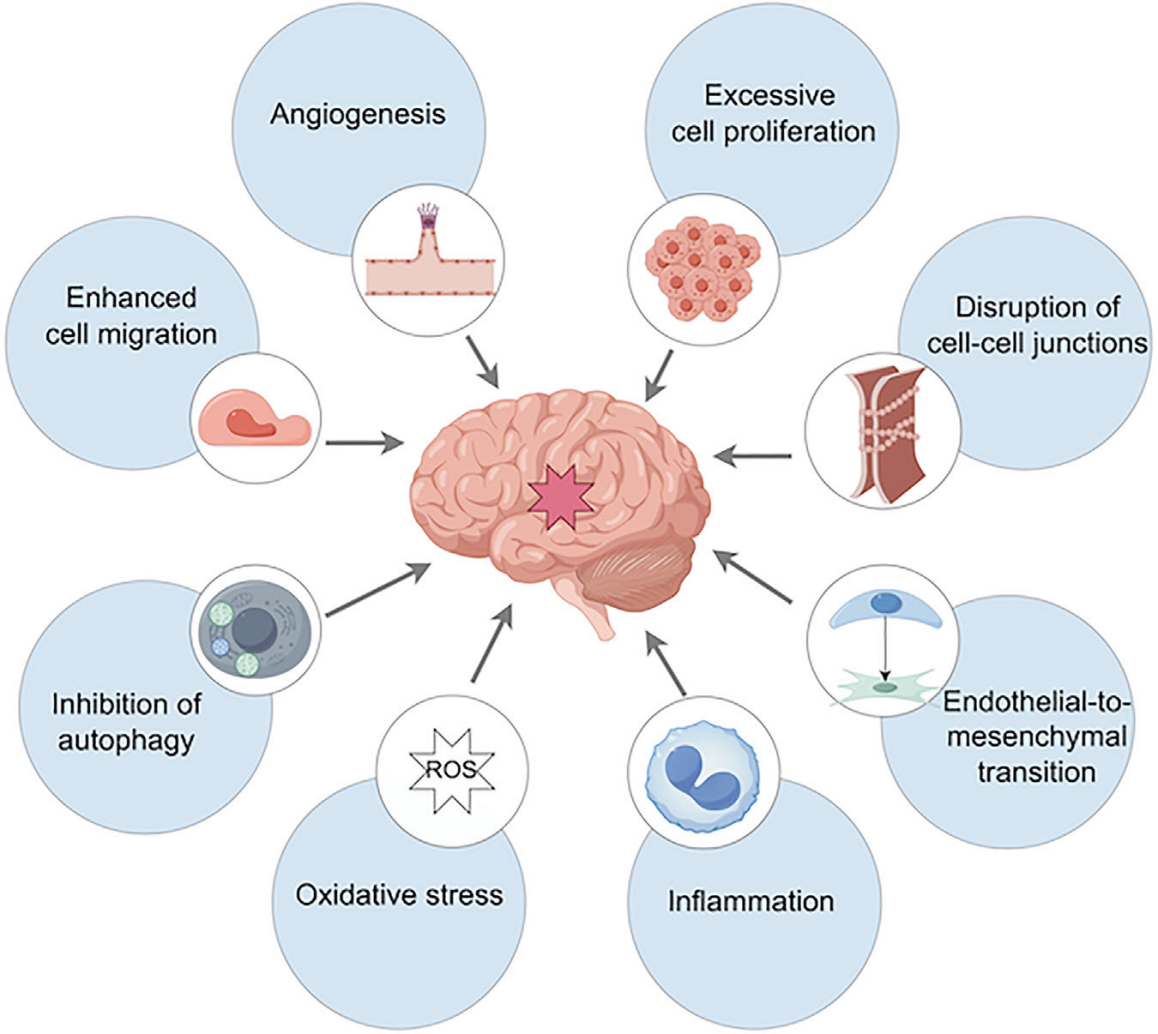
Schematic overview of the cellular and molecular features of cerebral cavernous malformation.

CCM is classified into familial and sporadic types (Smith 2024). Familial CCM often involves multiple lesions and a positive family history, while sporadic CCM generally features a single lesion (Figure 1) and no associated family history. Recent advances in genetic research have provided significant insights into the molecular and cellular phenotypes disrupted in CCM (Figure 2). Understanding the genetic mutations, including both germline and somatic mutations, is crucial for identifying potential therapeutic targets.

Due to progress in understanding the genetic basis of CCM, recent advancements have identified several potential therapeutic targets aimed at modulating the molecular pathways implicated in CCM pathogenesis. These include fasudil, an inhibitor that inhibits the mitogen‐activated protein kinase kinase kinase 3 (MAP3K3)–Kruppel‐like factor (KLF) signaling pathway, and rapamycin, an mammalian target of rapamycin (mTOR) inhibitor that inhibits the phosphatidylinositol 3‐kinase (PI3K)—protein kinase B (AKT)–mTOR pathway, and other agents (Zuurbier et al. 2022; L. Li et al. 2023; Shenkar et al. 2019, 2017). The identification of these therapeutic targets marks a significant advance in potential treatment options for CCM. By targeting the specific molecular and cellular pathways associated with CCM pathology, these emerging therapies are expected to improve clinical outcomes and reduce the burden of CCM.

This review provides an overview of the genetic factors driving CCM, highlighting the roles of both germline and somatic mutations. In addition, this review aims to examine the genetic and molecular basis of CCM and explore both current and emerging therapeutic strategies targeting the molecular pathways involved in its pathogenesis.

## 2 Gene Mutations and Their Related Pathogenesis in CCM

Heterozygous germline mutations in CCM1, CCM2, or CCM3 are the genetic causes of familial CCM (Dalyai et al. 2011; X. Li, Fisher, and Boggon 2015). These mutations follow an incomplete autosomal dominant pattern. Besides germline mutations, somatic mutations, including those in MAP3K3 and phosphatidylinositol‐4,5‐bisphosphate 3‐kinase catalytic subunit alpha (PIK3CA) within CCM lesions, have also been identified, suggesting a two‐hit mechanism driving CCM pathogenesis (Hong et al. 2021; J. Ren et al. 2023; Huo et al. 2023; A. Ren et al. 2021). Different germline mutations and somatic mutations in CCM cause different alterations in protein function and mediate corresponding biological functions in vivo, as shown in Table 1.

**TABLE 1:** Summary of pathogenic genes and relative functions in CCM.

Gene	Genetic locus	Interacted protein	Mutant cell	Changes in protein	Function
*CCM1*/*KRIT1*	7q21.2	Rap1 HEG1 ICAP1	Germline mutation	Loss of function	Cell adhesion Cell migration Autophagy Oxidative stress
*CCM2*	7p13	KRIT1 PDCD10 MAP3K3	Germline mutation	Loss of function	Cell–cell interactions Angiogenesis Lumen formation
*CCM3*/*PDCD10*	3q26.1	CCM2 STK25 Paxillin PTPN13 UNC13D	Germline mutation	Loss of function	Cell–cell junctions Cell migration Endothelial barrier Angiogenesis Autophagy
*MAP3K3*	17q23.3	CCM2 MEK5	Somatic mutation	Gain of function	Cell survival Cell proliferation Angiogenesis
*PIK3CA*	3q26.32	AKT mTOR	Somatic mutation	Gain of function	Cell proliferation Cell growth Angiogenesis

Abbreviations: AKT, protein kinase B; CCM, cerebral cavernous malformation; HEG1, heart of glass1; ICAP1, Integrin cytoplasmic domain‐associated protein‐1; KRIT1, Krev interaction trapped 1; MAP3K3, mitogen‐activated protein kinase kinase kinase 3; MEK5, mitogen‐activated protein kinase kinase 5; mTOR, mechanistic target of rapamycin.; PDCD10, programmed cell death 10; PIK3CA, Phosphatidylinositol‐4,5‐bisphosphate 3‐kinase catalytic subunit alpha; PTPN13, protein tyrosine phosphatase non‐receptor type 13; Rap1, Ras‐associated protein 1; STK25, Serine/threonine protein kinase 25; UNC13D, Unc‐13 Homolog D.

### 2.1 Germline Mutations

#### 2.1.1 CCM1 (KRIT1)

In 1999, several studies demonstrated that *KRIT1* gene mutation, located on chromosome 7q, can cause CCM, thereby establishing the *KRIT1* gene as the first pathogenic gene for familial CCM (Sahoo et al. 1999; Laberge‐le Couteulx et al. 1999). *KRIT1* gene mutations are found in about 60% of familial CCM, with more than 100 mutation sites identified to date (Akers et al. 2017; Ricci et al. 2021). The *KRIT1* gene encodes the KRIT1 protein. Serebriiskii et al. (1997) discovered the KRIT1 protein in 1997 through a yeast two‐hybrid screen for Ras‐proximate‐1 (Rap1)‐interacting proteins. This protein, also known as Kirsten‐ras‐revertant 1 (Krev‐1), is classified within the Ras family of small GTPases (Serebriiskii et al. 1997). KRIT1 can form a ternary complex with both Rap1 and heart of glass homolog 1 (HEG1), which is crucial for cardiovascular development (Gingras, Puzon‐McLaughlin, and Ginsberg 2013).

Deficiency of the *KRIT1* gene leads to instability of integrin cytoplasmic domain‐associated protein‐1 (ICAP1), which promotes the activation of β1 integrin, resulting in increased ras homolog family member A (RhoA)–dependent contraction of the extracellular matrix and destabilization of intercellular junctions, thus contributing to the progression of CCM lesions (W. Liu et al. 2013; Faurobert et al. 2013). KRIT1 is also crucial for maintaining cellular homeostasis and protecting against oxidative stress, particularly through processes such as autophagy and antioxidant signaling (Retta et al. 2020; Antognelli et al. 2020). The *KRIT1* gene might also be crucial in the function of the intestinal epithelium. Y. Wang et al. (2019) found that the intestinal epithelium expresses KRIT1, which contribute in maintaining the barrier function of intestinal epithelial cells. KRIT1 deficiency increases tight junction permeability, tumor necrosis factor–induced apoptosis, and intestinal epithelial barrier loss.

A total of 135 pathogenic variants of the *KRIT1* gene have been identified to date. These encompass a range of variant types, with 61 single nucleotide variants representing the most prevalent. Subsequently, there are 40 deletion variants, 20 duplication variants, 3 insertion–deletion variants, 4 insertion variants, and 7 microsatellite variants. Mutations of varying types have resulted in corresponding molecular consequences, with the highest number of shifted‐code mutations at 65. This was followed by 55 nonsense mutations, 13 splice‐site mutations, and 2 missense mutations.

Familial CCM patients with KRIT1 mutations are more likely to have extra‐brain manifestations, such as vertebral hemangiomas (Clatterbuck et al. 2002), hepatic angiomas (Toldo et al. 2009), and cutaneous vascular malformations (Sirvente et al. 2009).

#### 2.1.2 CCM2 (MGC4607)

Denier et al. (2004) and Liquori et al. (2003) identified the *CCM2* gene, also known as MGC4607, located on chromosome 7p, as the second pathogenic gene associated with familial CCM. The CCM2 gene, consisting of 10 coding exons, encodes the CCM2 protein, which features a phosphotyrosine binding (PTB) domain and is primarily localized in pyramidal neurons, endothelial cells, and astrocytes in the cerebral cortex (Seker et al. 2006). The interaction between CCM1, CCM2, and CCM3 results in a trimeric complex, where CCM2 functions as the main intermediary between CCM1 and CCM3 (Zawistowski et al. 2005; Oldenburg et al. 2021).

CCM2 is mainly localized not only in the cytoplasm but also found in the nucleus (J. Zhang et al. 2007). Around 20% of familial CCM result from *CCM2* gene mutations leading to functional loss (de Vos et al. 2017). Patients with CCM2 mutations often experience fewer clinical symptoms and present with a less severe phenotype compared to those with other *CCM* gene mutations (Denier et al. 2006).

CCM2 combines with CCM3 to create a complex involving serine/threonine kinase 25 (STK25) (Voss et al. 2007). CCM2 is involved in regulating endothelial cytoskeletal framework, cell–cell interactions, and lumen formation (Whitehead et al. 2009). It is crucial for angiogenesis, as the constitutive loss of CCM2 leads to early embryonic lethality, and the loss of endothelial CCM2 results in defects in major arterial and venous vessels (Boulday et al. 2009). Moreover, the zebrafish counterpart of human CCM2 is crucial for the development of myocardial tissue (Mably et al. 2006). Depletion of CCM2 activates RhOA GTPases, causing the development of CCM lesions. Simvastatin, a medication that targets and inhibits Rho GTPases, has been shown to rescue the cellular phenotype in mice (Whitehead et al. 2009). CCM2 also directly interacts with MAP3K3 (Uhlik et al. 2003), and the loss of CCM2 activates the downstream MAP3K3–KLF2/4 signaling pathway (Cullere et al. 2015; Zhou et al. 2016), leading to CCM lesions.

Thirty‐two pathogenic variants have been identified in the CCM2 gene, encompassing a range of variant types. Among them, single‐nucleotide variants are the most frequent, with 12 identified. This is followed by 11 deletion variants, 6 duplication variants, 1 insertion–deletion variant, 1 insertion variant, and 1 microsatellite variant. These different mutation types lead to various molecular consequences, with frameshift mutations being the most common, accounting for 16. In addition, 11 nonsense mutations, 4 splice‐site mutations, and 1 missense mutation have also been observed.

#### 2.1.3 CCM3 (PDCD10)

Bergametti et al. (2005) identified the *CCM3* gene, also known as *PDCD10*, located on chromosome 3q25.2‐27, as the third pathogenic gene associated with familial CCM. CCM3 not only binds to CCM2 but also is frequently observed in the striatin‐interacting phosphatase and kinase (STRIPAK) complex (Hilder et al. 2007). Loss of CCM3 disrupts the STRIPAK signaling pathway (Lant et al. 2015; Goudreault et al. 2009). In addition, CCM3 interacts with various proteins, including paxillin (X. Li et al. 2011), PTPN13 (Voss et al. 2007), and UNC13D (Y. Zhang et al. 2013). CCM3 mutations account for approximately 10%–40% of familial CCM (Q. Wang 2005; Riant et al. 2010). In contrast to CCM1 and CCM2 mutations, CCM3 mutations often result in earlier onset, more severe symptoms, and a heightened risk of cerebral hemorrhage (Denier et al. 2006; Fauth et al. 2015; Shenkar et al. 2015; Cigoli et al. 2014). This suggests that the pathogenic mechanisms underlying CCM3 may differ from those associated with CCM1 and CCM2, involving unique pathways such as the impairment of the colonic mucosal barrier, which significantly influences the disease course (Tang et al. 2019). Loss of CCM3 is also linked with senescence (Guerrero et al. 2015) and an increased risk of multiple meningiomas (Riant et al. 2013).

CCM3 is vital for regulating tight junction and brain endothelial barrier permeability (Stamatovic et al. 2015). Loss of CCM3 activates the MAP kinases extracellular signal‐regulated kinase (ERK) 1/2, leading to increased phosphorylation and degradation of cortactin. Blocking ERK1/2 prevents the disassembly of tight junction complexes and the resulting increase in brain endothelial barrier permeability caused by CCM3 loss (Stamatovic et al. 2015). CCM3 is also crucial for angiogenesis; it targets delta like 4 (DLL4) in endothelial cells, and its loss inhibits the DLL4–NOTCH signaling pathway, promoting angiogenesis (You et al. 2013). He et al. (2010) found that mice lacking CCM3, which stabilizes vascular endothelial growth factor receptor 2 (VEGFR2), exhibit impaired VEGFR2‐mediated signaling in endothelial cells and during embryogenesis, resulting in impairments in embryonic angiogenesis and premature mortality. Zhou et al. (2016) used a newborn mouse CCM model to show that CCM3, in combination with CCM1 and CCM2, forms a complex with MAP3K3, activating the MAP3K3–KLF2/4 signaling pathway and mediating CCM pathogenesis.

Beyond its involvement in cell–cell junctions and angiogenesis, CCM3 contributes to CCM pathogenesis through other mechanisms. CCM3 is vital for neurovascular development (Louvi et al. 2011). Loss of CCM3 in neurons activates astrocytes via the AKT signaling pathway, causing cell‐autonomous effects and nonautonomous effects such as diffuse vascular dilation and CCM‐like lesions (Louvi et al. 2011). Marchi et al. (2015) showed that loss of CCM3 function impairs autophagy by upregulating the rapamycin pathway, leading to abnormal protein aggregation. Maddaluno et al. (2013) found that mice with CCM3 knockout exhibit endothelial‐to‐mesenchymal transition (EndMT) as a result of transforming growth factor (TGF)‐beta signaling, and inhibiting TGF‐beta can reduce CCM lesions by preventing this transition. L. Chen et al. (2009) found that CCM3 promotes apoptosis in vitro cell models, and its loss may induce CCM lesions by causing abnormal cell apoptosis in the neurovascular unit. *CCM3* gene mutation can also lead to Golgi dysfunction, abnormal cell migration (Fidalgo et al. 2010), and disruption of interactions between endothelial and pericytes (Min and Zhou 2023).

Thirty‐nine pathogenic variants have been identified in the *PDCD10* gene, representing a diverse array of variant types. Deletion variants are the most common, accounting for 15, followed by 14 single‐nucleotide variants, 7 repeat variants, 2 microsatellite variants, and 1 insertion variant. These mutations result in various molecular consequences, with frameshift mutations being the most frequent, totaling 23. In addition, there are 8 nonsense mutations and 8 splice‐site mutations.

### 2.2 Somatic Mutation

Gene mutations in somatic cells are inherently unstable, contributing to processes such as cancer and aging (Vijg 2014; García‐Nieto, Morrison, and Fraser 2019). These genetic mutations are present in all tissues and accumulate with age, resulting in the proliferation of cell clones in both malignant and benign tissues (Kakiuchi and Ogawa 2021; Martincorena 2019; Jolly and Van Loo 2018). Recent investigations using high‐throughput sequencing on surgical samples from sporadic CCM patients have revealed somatic mutations in the *PIK3CA* and *MAP3K3* genes within CCM lesions (Hong et al. 2021; Weng et al. 2021; Peyre et al. 2021). In contrast, mutations in the three established *CCM* genes are infrequently observed in sporadic CCM.

#### 2.2.1 MAP3K3

The gene *MAP3K3* on chromosome 17q23.3 encodes MAP3K3, a polypeptide comprising 626 amino acids polypeptide (Ellinger‐Ziegelbauer et al. 1997). MAP3K3 functions as a critical node in the mitogen‐activated protein kinase (MAPK) pathway by phosphorylating extracellular signal‐regulated kinase 5 (MEK5), which activates extracellular signal‐regulated protein kinase 5 (ERK5) to influence cell survival, proliferation, and angiogenesis (Drew, Burow, and Beckman 2012). CCM2 and MAP3K3 interact directly to maintain vascular integrity and permeability through the control of the Rho/Rho‐associated coiled‐coil containing protein kinase (ROCK) pathway (Fisher et al. 2015). Previous studies have identified toll‐like receptor 4 (TLR4) (Tang et al. 2017) and CDC42 (Castro et al. 2019) as upstream targets of MAP3K3. The CCM protein complex acts as an inhibitor of MAP3K3 (Zhou et al. 2015). CCM deficiency activates the MAP3K3 signaling axis, increases the expression of transcription factors KLF2/4, and further activates the downstream RhoA–ROCK signaling pathway (Fisher et al. 2015; Tang et al. 2017).

Hong et al. (2021) conducted whole‐exome sequencing on lesion samples from 81 sporadic CCM patients and first confirmed that MAP3K3 somatic mutations represent the core genetic mechanism underlying sporadic CCM. Previous studies have indicated that MAP3K3 mutations are less frequently associated with bleeding events compared to *CCM* gene mutations, which exhibit a different phenotype (Huo et al. 2022). One possible explanation for this discrepancy is that MAP3K3 mutations result in less damage to the blood–brain barrier and less accumulation of local anticoagulant molecules in endothelial cells than *CCM* gene mutations (Huo et al. 2022).

Snellings et al. (2022) found that *MAP3K3* and the known *CCM* genes are mutually exclusive. This implies that a mutation in *MAP3K3* or three *CCM* genes can independently cause sporadic CCM, without the need for additional mutations in any of the other genes. The study further identified that MAP3K3 mutation leads to sporadic rather than familial CCM and can co‐occur with PIK3CA mutation. Moreover, it proposed a molecular genetic explanation for the frequent association between developmental venous anomaly (DVA) and sporadic CCM. Specifically, DVA is driven by PIK3CA mutation and these DVA cells provide the molecular template for secondary somatic mutation in MAP3K3, ultimately resulting in the formation of sporadic CCM. This interesting hypothesis offers a novel perspective on the mechanism underlying sporadic CCM and warrants further study for validation. Huo et al. (2023) demonstrated that MAP3K3 gain‐of‐function mutation can cause CCM lesions in mice through adeno‐associated virus vectors (Huo et al. 2023). In addition, J. Ren et al. (2023) found that MAP3K3 gain‐of‐function mutation can independently stimulate the mTOR signaling pathway, thereby causing CCM in mice, which mirrored human sporadic CCM in pathology.

#### 2.2.2 PIK3CA

PIK3CA is essential to angiogenesis and vascular development (Graupera et al. 2008). PIK3CA gain‐of‐function mutation activates the PI3K–AKT signaling pathway, triggering oncogenic effects (Lai, Killingsworth, and Lee 2015). These mutations drive cell proliferation, growth, metabolism, angiogenesis, and metastasis (Polo et al. 2015; Herberts et al. 2020), resulting in PIK3CA‐associated overgrowth spectrum disorders (Madsen, Vanhaesebroeck, and Semple 2018; Keppler‐Noreuil et al. 2015). Beyond functioning as oncogenes in various cancers (Polo et al. 2015; Samuels et al. 2004; Zhao et al. 2005), PIK3CA gain‐of‐function mutations also play a role in the pathogenic mechanisms of several vascular malformations (Le Cras and Boscolo 2019; Le Cras et al. 2020).

In addition to sporadic CCM, PIK3CA gain‐of‐function mutation is also found in three genotypes of familial CCM (Hong et al. 2021; A. Ren et al. 2021; Weng et al. 2021). In sporadic CCM, *CCM* gene mutation frequently co‐occur with PIK3CA gain‐of‐function mutation (Weng et al. 2021). The incidence of PIK3CA gain‐of‐function mutations in sporadic CCM is higher than that of *CCM* gene mutations (McDonald et al. 2014). Creating mouse models with this mutation is challenging, likely due to embryonic lethality (Castel, Rauen, and McCormick 2020), which results from angiogenesis abnormalities and severe vascular malformations during early embryonic development (Schonning et al. 2021; Hare et al. 2015; Luks et al. 2015).

A recent study revealed a multigene “triple hit” mechanism in CCM (A. Ren et al. 2021). Like cancer, CCM genes act as vascular “suppressor genes” inhibiting blood vessel growth, while PIK3CA functions as a vascular “oncogene” promoting excessive blood vessel growth. Research has indicated that knocking out CCM genes results in the development of CCM in mice during the early postnatal period, and PIK3CA mutations induce CCM development in adult mice (A. Ren et al. 2021; McDonald et al. 2011; Snellings et al. 2021).

## 3 The Potential Pharmacotherapy Targeting Various Pathways

**FIGURE 3:**
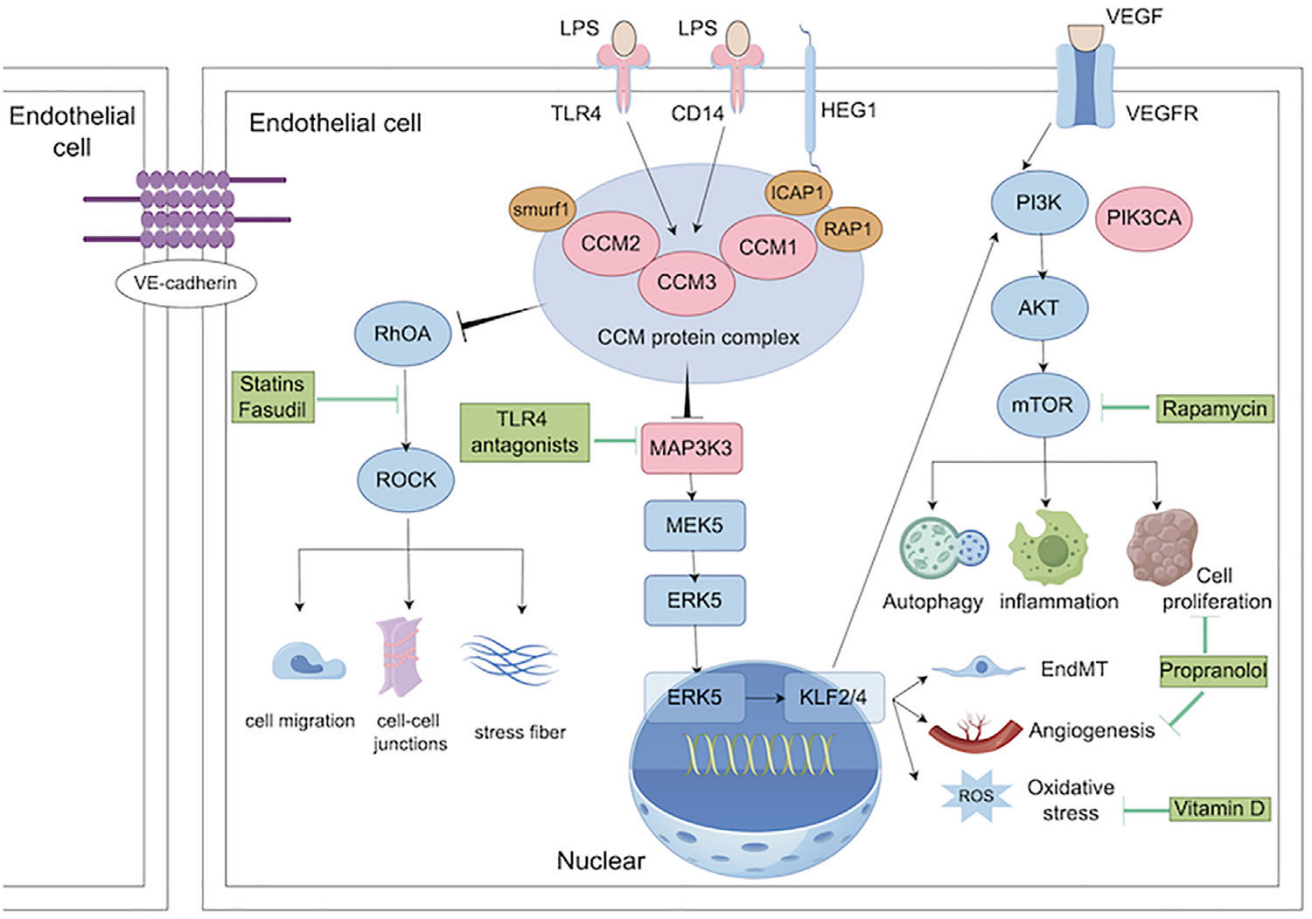
Key signaling pathways in the pathogenesis of CCM. CCM1, CCM2, and CCM3 encode proteins that form the CCM protein complex, which plays a critical role in vascular stability. The complex's upstream regulation is influenced by the interaction with LPS through binding to TLR4 and CD14. In familial CCM, loss‐of‐function mutations in the CCM gene deregulate the inhibitory effect on downstream RhoA, which in turn activates the RhoA–ROCK signaling pathway and promotes cell migration, deletion of intercellular junctions, and stress fiber formation. Moreover, loss‐of‐function mutations in the CCM genes or somatic gain‐of‐function mutations in the MAP3K3 gene activate the MAP3K3–KLF2/4 signaling pathway, which drives EndMT, angiogenesis, and oxidative stress. In addition, somatic gain‐of‐function mutations in PIK3CA lead to activation of the PI3K–AKT signaling pathway, resulting in abnormal cell proliferation, increased inflammation, and impaired autophagy.

The causative genes for familial CCM include CCM1, CCM2, and CCM3, while for sporadic CCM, PIK3CA and MAP3K3 have been identified, with additional causative genes likely to be discovered in future studies. Mutations in different genes contribute to CCM pathogenesis by regulating distinct signaling pathways, which in turn mediate various cellular and molecular phenotypes (Figure 3). Consequently, therapeutic agents have been developed to target specific signaling pathways and their associated intervention points. The following sections will introduce the potential pharmacotherapy that have been developed for each of these signaling pathway intervention targets, as shown in Table 2.

**TABLE 2:** Compounds or drugs in preclinical or clinical studies for the treatment of CCM.

Therapeutic strategy	Compound or drug	Targeted molecular pathway	Mechanisms	Model	Lesion pathogenesis	Approved application
RhoA/Rho kinase inhibitor	Statins	RhoA–ROCK	Regulate cell migration, proliferation, adhesion, and apoptosis	Murine Human	Chronic bleeding	Antihyperlipidemic drug
	Fasudil	RhoA–ROCK	Regulate cell migration, proliferation, adhesion, and apoptosis	Murine	Lesion burden Chronic bleeding Long‐term survival	Cerebral vasospasm
mTORC1 inhibitor	Rapamycin	PI3K–AKT–mTOR	Regulate cell growth, apoptosis, and autophagy	Murine	Lesion growth Chronic bleeding	Immunosuppressive drug Antitumor drugs
Angiogenesis inhibitors	Propranolol	β‐Adrenoceptor receptor	Promotes apoptosis, inhibits angiogenesis, cell proliferation and migration.	Murine Human	Lesion growth Lesion burden Chronic bleeding	Antihypertensive drug
Antioxidant drugs	Vitamin D	Inflammation	Antioxidant, anti‐inflammatory, autophagy‐promoting, and endothelial‐stabilizing	Murine Human	Lesion burden Chronic bleeding	Vitamin D deficiency osteoporosis
Toll‐like receptor 4 antagonists	TAK242 (Resatorvid)	MAP3K3–KLF	Inhibition of EMT	Murine Human	Lesion number Lesion burden Lesion progression	Sepsis

Abbreviations: AKT, protein kinase B; CCM, cerebral cavernous malformation; EMT, epithelial‐mesenchymal transition.; KLF, Kruppel‐like factor; MEKK3, mitogen‐activated protein kinase kinase kinase 3; mTOR, mechanistic target of rapamycin; mTORC1, mechanistic target of rapamycin complex 1; PI3K, Phosphoinositide 3‐kinase; RhoA, ras homolog family member A; ROCK, Rho‐associated coiled‐coil containing protein kinase.

### 3.1 RhoA/Rho Kinase Inhibitor

ROCK is a serine/threonine protein kinase that operates as a downstream mediator of the RhoA GTPase (Julian and Olson 2014). Several key cellular processes are significantly regulated by the RhoA–ROCK signaling pathway, including cell migration, proliferation, differentiation, and apoptosis (Sawma et al. 2022; Riento and Ridley 2003). The CCM complex inhibits RhoA/ROCK signaling and mutations in *CCM* genes activate the RhoA–ROCK pathway, leading to the formation of CCM lesions (Richardson et al. 2013; Stockton et al., 2010). Therefore, inhibiting RhoA/ROCK signaling might serve as a potential strategy to slow or prevent the progression of CCM lesions.

#### 3.1.1 Statins

As indirect ROCK inhibitors, statins have shown promise in reducing CCM lesion burden in animal models. Studies have demonstrated that atorvastatin (80 mg/kg/d) significantly reduces CCM lesion burden in PDCD10^+/−^Trp53^−/−^and PDCD10^+/−^Msh2^−/−^mice (Shenkar et al. 2019). In CCM1^+/−^Msh2^−/−^, CCM2^+/−^Trp53^−/−^, PDCD10^+/−^Trp53^−/−^, and PDCD10^+/−^Msh2^−/−^mice, simvastatin reduced chronic bleeding of CCM lesions but did not improve survival or reduce lesion burden (Shenkar et al. 2019, 2017).

However, evidence suggests that while statins show promise in animal studies, their therapeutic impact on CCM patients may be less substantial. Although a study found that compared to the use of antiplatelet therapy alone, the combination of statins and antiplatelet agents significantly reduced the risk of hemorrhage in CCM patients, which suggests a potential synergistic effect between statins and antiplatelet therapy (Marques et al. 2023). However, several researches have failed to demonstrate a correlation between statin therapy and a lower incidence of CCM bleeding (Zuurbier et al. 2022; Previch et al. 2022; Santos et al. 2022; Wildi et al. 2023; B. Chen et al. 2022) or brain permeability (Mabray et al. 2020). Furthermore, research demonstrated that statins are ineffective in reducing the risk of focal neurological deficits, including headaches, epilepsy, and sensorimotor disorders in CCM patients (Zuurbier et al. 2022; Wildi et al. 2023).

Given the discrepancies in the efficacy of statins observed in CCM animal models and patients, more randomized controlled trials are required to better understand the therapeutic effect of statins in managing CCM.

#### 3.1.2 Fasudil

As another ROCK inhibitor, fasudil has been demonstrated to have therapeutic effects on CCM in multiple preclinical studies. Previous studies demonstrated that fasudil intervention (100 mg/kg/d) in CCM1^+/−^Msh2^−/−^, CCM2^+/−^Trp53^−/−^, PDCD10^+/−^Trp53^−/−^, and PDCD10^+/−^Msh2^−/−^mice resulted in a significant reduction in CCM lesion burden, a reduction in chronic bleeding, and improved long‐term survival rates (Shenkar et al. 2019, 2017; McDonald et al. 2012).

### 3.2 mTORC1 Inhibitor

mTOR is critical in regulating cell growth, apoptosis, autophagy, and multiple other cellular activities (G. Liu and Sabatini 2020; Robles‐Flores, Moreno‐Londoño, and Castañeda‐Patlán 2021). The mTOR signaling pathway consists mainly of two complexes: mTOR complex 1 (mTORC1) and mTOR complex 2 (mTORC2) (Foster and Fingar 2010). While mTORC1 is chiefly responsible for regulating cell growth and metabolism and is responsive to rapamycin, mTORC2 focuses on cell survival, proliferation, and cytoskeletal changes, exhibiting limited responsiveness to rapamycin (Kim et al., 2017; Saxton and Sabatini 2017). The phosphorylation and activation of mTOR are influenced by several upstream signaling proteins in the PI3K–AKT–‐mTOR pathway (Karki et al., 2016). Studies have shown that KRIT1 loss‐of‐function mutation activate mTOR.

### 3.3 Rapamycin

A. Ren et al. (2021) discovered that an acquired mutation in the oncogene PIK3CA leads to the progression of CCM lesions, implying that CCM progression is related to enhanced mTORC1 activity. Furthermore, in a mouse model, it was verified that the growth of CCM can be effectively reduced by the mTORC1 inhibitor rapamycin. In sporadic CCM caused by *MAP3K3* gene mutations, the mTOR pathway is significantly activated, leading to endothelial expansion and impairment of the blood–brain barrier (J. Ren et al. 2023). In mice with MAP3K3‐induced CCM, rapamycin has been shown to reduce the bleeding phenotype (J. Ren et al. 2023). These results indicate that rapamycin could be effective in reducing lesions in sporadic CCM.

Although rapamycin has shown efficacy in sporadic CCM with PIK3CA gain‐of‐function mutation, its therapeutic effect in familial CCM is not significant and may even be harmful. In a previous study, Alcazar‐Felix et al. (2024) used a familial CCM murine model with targeted deletion of *CCM* genes in endothelial cells to investigate the role of rapamycin in CCM lesion progression and bleeding. They found that increasing rapamycin doses were associated with greater CCM lesion burden. The therapeutic effect of rapamycin in familial CCM requires further investigation.

### 3.4 Angiogenesis Inhibitors

#### 3.4.1 Propranolol

Propranolol, a beta‐blocker, was initially used to treat CCM based on its efficacy in managing infantile hemangiomas (Prey et al. 2016; Drolet et al. 2013; Léauté‐Labrèze et al. 2015), where it has been shown to attenuate these vascular lesions. Propranolol exerts its therapeutic effects on capillary malformations through several pathophysiological processes, including stimulating vasoconstriction (Léauté‐Labrèze et al. 2008), promoting capillary endothelial cell apoptosis (Léauté‐Labrèze et al. 2008), inhibiting angiogenesis (Annabi et al. 2009), and inhibiting endothelial cell proliferation and migration (Lamy et al. 2010).

Several studies have since found that propranolol also has therapeutic effects on CCM. Moschovi et al. 2010) first reported a case of propranolol treatment for a giant CCM in an infant, where the lesion significantly decreased in size after 10 days of receiving 2 mg/kg of propranolol. Filippidis et al. (2011) proposed propranolol as an ideal candidate drug for CCM treatment. Following these initial reports, several case studies have suggested that propranolol can prevent lesion growth and cerebral hemorrhage in CCM patients (Berti et al. 2014; Hoffman et al. 2020; Zabramski et al. 2016). A large randomized controlled trial demonstrated the safety and efficacy of propranolol in the treatment of CCM, which showed that propranolol could significantly reduce the incidence of clinical events in CCM patients, highlighting its potential as a therapeutic agent (Lanfranconi et al. 2023). In addition, another study found that CCM patients taking propranolol were associated with a lower prevalence of depression, suggesting potential benefits beyond the management of hemorrhage and focal neurological deficits (Meessen et al. 2024). However, several other clinical studies have not found a significant correlation between propranolol administration and a decreased risk of cerebral hemorrhage or neurological deficits in CCM patients (Previch et al. 2022; Santos et al. 2022; B. Chen et al. 2022; Goldberg et al. 2018). A meta‐analysis that included five clinical studies on propranolol's therapeutic effects did not find significant efficacy in preventing cerebral hemorrhage or focal neurological deficits in CCM patients (Ikramuddin et al. 2023). This lack of meaningful results may be due to the few CCM cases available and the insufficient number of high‐quality clinical trials.

Several preclinical studies have also highlighted the efficacy of propranolol in treating CCM. Both Oldenburg et al. (2021) and W. Li et al. (2021) have shown that propranolol significantly decreases the size and severity of CCM lesions in murine models. W. Li et al. (2021) suggested that propranolol treats CCM by blocking β‐1 receptors, whereas Oldenburg et al. (2021) demonstrated that the protective role of propranolol on pericytes leads to better vascular stability.

Despite the lack of clear efficacy in some cohort and case‐control studies, preclinical studies and studies in infantile hemangiomas suggest the therapeutic potential of propranolol in CCM. To confirm this potential, additional comprehensive randomized controlled trials will be necessary.

### 3.5 Antioxidant Drugs

#### 3.5.1 Vitamin D

Numerous studies suggest that autophagy dysfunction and redox imbalance are key contributors to the pathogenesis of CCM lesions (Marchi et al. 2016; Retta and Glading 2016). Evidence indicates that endothelial cell dysfunction is linked to vitamin D deficiency in both healthy individuals and those with various medical conditions (Yiu et al. 2011; Al Mheid et al. 2011). Vitamin D exhibits antioxidant properties in endothelial cells and may prevent cell death by modulating the interplay between apoptosis and autophagy (Uberti et al. 2014).

A correlation exists between blood vitamin D levels and the severity of CCM, being significantly lower in patients with more aggressive forms of CCM, such as those with early onset, symptomatic bleeding, and a high lesion burden (Girard et al. 2016). Clinical studies have shown that vitamin D supplementation is associated with a reduced risk of hemorrhage in CCM patients (Previch et al. 2022). Other prospective studies have indicated that CCM patients with intracerebral hemorrhage have lower vitamin D levels compared to those without hemorrhage (Flemming et al. 2020a, 2020b). Single nucleotide polymorphism (SNP) in genes associated with vitamin D metabolism were found to correlate with CCM disease severity in a genome‐wide association study (Choquet et al. 2016).

As a compound with antioxidant, anti‐inflammatory, autophagy‐promoting, and endothelial‐stabilizing properties, vitamin D has been shown in mouse models of CCM to reduce lesion burden (Retta and Glading 2016; Marchi, Retta, and Pinton 2016; Gibson et al. 2015). However, some preclinical studies have yielded negative results, with vitamin D failing to significantly reduce CCM lesion loads (Detter et al. 2020). To fully understand the therapeutic potential of vitamin D in CCM, further research is essential.

### 3.6 Toll‐Like Receptor 4 Antagonists

Familial CCM patients with the same mutation often exhibit clinical phenotypes of varying severity, even within the same family (Denier, Labauge, et al. 2004; Choquet, Nelson, et al. 2014). This variability may be due to genetic modifiers or acquired disease factors. Choquet, Pawlikowska, et al. (2014) found that multiple SNP loci, including TLR4, were significantly associated with the severity of CCM, including lesion number and burden, suggesting that these SNP loci are genetic regulators of CCM. Subsequent clinical and preclinical studies identified TLR4 as a key stimulator of CCM formation (Tang et al. 2017; Starke et al. 2017), providing a new target for CCM treatment. CCM patients with more polymorphisms in the *TLR4* gene tend to have a greater lesion burden. The presence of Gram‐negative bacteria or lipopolysaccharide stimulates TLR4, accelerating CCM development in mice, while blocking TLR4 prevents CCM formation (Tang et al. 2017).

Additionally, a relationship between TLR4 activity and the progression of CCM lesions has been found, explaining the more severe clinical phenotype in patients with CCM3 mutations (Tang et al. 2019). CCM3‐deficient mice exhibit significantly elevated blood TLR4 activity and disruption of the intestinal epithelial barrier, which stimulates the progression of CCM foci through the gut–brain axis (Tang et al. 2019).

## 4 Conclusions and Perspectives

In summary, the progress in genetic research has revealed the important roles of germline and somatic mutations in CCM development. Familial CCM is predominantly linked to germline mutations in KRIT1, CCM2, and PDCD10, whereas recent evidence highlights the importance of somatic mutations in the initiation and progression of these lesions. Understanding the genetic landscape of CCM has revealed the molecular mechanisms underlying lesion formation and identified critical pathways for therapeutic intervention. The disruption of cellular signaling and endothelial integrity due to these genetic alterations provides a foundation for developing targeted therapies.

Further research is needed to better understand the genetic and molecular foundations of CCM, exploring the roles of additional genetic variants and epigenetic factors that may contribute to disease variability and progression. Approaches such as small molecule inhibitors and gene therapy show great promise. In summary, despite substantial advances in elucidating the genetic mechanisms and identifying therapeutic targets for CCM, continued research is essential. Large, multicenter studies will be crucial for confirming these results and applying them to clinical practice.

## Author Contributions

**Zhuangzhuang Zhang**: conceptualization, investigation, visualization, writing–original draft. **Jianwen Deng**: data curation, methodology, writing–review and editing. **Weiping Sun**: supervision, funding acquisition, writing–review and editing. **Zhaoxia Wang**: supervision, funding acquisition, writing–review and editing.

## Ethics Statement

The authors have nothing to report.

## Conflicts of Interest

The authors declare no conflicts of interest.

### Peer Review

The peer review history for this article is available at https://publons.com/publon/10.1002/brb3.70223

## Data Availability

The authors have nothing to report.
